# High-throughput transformation of *Saccharomyces cerevisiae* using liquid handling robots

**DOI:** 10.1371/journal.pone.0174128

**Published:** 2017-03-20

**Authors:** Guangbo Liu, Clayton Lanham, J. Ross Buchan, Matthew E. Kaplan

**Affiliations:** 1 Department of Molecular and Cellular Biology; University of Arizona, Tucson, Arizona, United States of America; 2 Functional Genomics Core facility, University of Arizona, Tucson, Arizona, United States of America; CNR, ITALY

## Abstract

*Saccharomyces cerevisiae* (budding yeast) is a powerful eukaryotic model organism ideally suited to high-throughput genetic analyses, which time and again has yielded insights that further our understanding of cell biology processes conserved in humans. Lithium Acetate (LiAc) transformation of yeast with DNA for the purposes of exogenous protein expression (e.g., plasmids) or genome mutation (e.g., gene mutation, deletion, epitope tagging) is a useful and long established method. However, a reliable and optimized high throughput transformation protocol that runs almost no risk of human error has not been described in the literature. Here, we describe such a method that is broadly transferable to most liquid handling high-throughput robotic platforms, which are now commonplace in academic and industry settings. Using our optimized method, we are able to comfortably transform approximately 1200 individual strains per day, allowing complete transformation of typical genomic yeast libraries within 6 days. In addition, use of our protocol for gene knockout purposes also provides a potentially quicker, easier and more cost-effective approach to generating collections of double mutants than the popular and elegant synthetic genetic array methodology. In summary, our methodology will be of significant use to anyone interested in high throughput molecular and/or genetic analysis of yeast.

## Introduction

*Saccharomyces cerevisiae* (Yeast) is a widely studied and highly utilized eukaryotic model organism, ideally suited to high throughput genetic analysis. Key reasons for this include yeast’s ability to exist in a haploid state, allowing more direct genotype-phenotype analyses, and efficient homologous recombination that allows easy editing of genomic sequence. Given this, many yeast genomic libraries are currently available, including epitope tagged ORF collections (GFP, TAP-tag etc.), as well as gene deletion, conditional expression, and over-expression libraries [1–5]. Among the approximate 6000 genes in the yeast genome, >31% are conserved between yeast and human species, most of which function in core aspects of eukaryotic cell function [6,7]. For example, Nobel prize-winning studies in yeast have been fundamental to our understanding of the mechanism and regulation of the cell cycle [8,9], secretion [10,11] telomere biology [12] and autophagy [13]. Yeast is therefore a powerful model for understanding basic cell biology.

Yeast has also proven itself a valuable model in the study of human disease. For instance, screening for suppressors or enhancers of toxicity associated with heterologous expression of mutant forms of human disease genes is increasingly common [14,15]. For example, expression in yeast of the human RNA binding protein TDP-43, which is associated with amyotrophic lateral sclerosis (ALS) and frontal temporal dementias, recapitulates the pathological phenotype of cytoplasmic aggregates and cellular toxicity, and lead to the identification of Ataxin-2 as a risk factor in human patients [16–19]. Furthermore, >20% of human disease genes have yeast homologs [20]. Yeast is therefore an attractive model to perform initial genetic and mechanistic analyses of various human diseases, particularly those that impact core cellular processes.

Synthetic Genetic Array analysis (SGA) is an elegant high-throughput method that allows synthetic genetic interactions to be assessed by generation of combinatorial mutant yeast strains via a systematic mating approach, followed by analyses of growth phenotypes [21,22]. Screening for enhancers or suppressors of toxicity associated with expression of human disease proteins, including TDP-43, has also utilized SGA methods [18,19]. In a typical SGA experiment, a query strain is systematically mated to a library of yeast gene deletion strains to generate an array of combinatorial mutant strains for analysis. Although a powerful method, SGA involves numerous selection steps, prior generation of a query mutant strain compatible with the SGA selection steps and takes approximately 18 days from start to finish. An alternative and potentially quicker means to generate libraries of yeast combinatorial mutants would be to transform strains from the same yeast gene deletion library with a plasmid or PCR product to directly express, delete or modify the query gene of interest in yeast gene deletion strains of interest. Description of such a method is currently lacking.

Lithium acetate based transformation [23,24] is a core yeast methodology, given its use in the expression of exogenous DNA via plasmid vectors, and homologous recombination-based modification of the genome with PCR-amplified DNA. Notably, a yeast transformation protocol for microtiter plate based transformation has been previously reported [23–25], but this still required significant manual handling steps. In this study, we expand on and further optimize these methods for use with liquid handling robotic systems now common in university and industrial settings. Our method is highly efficient, allowing 1200 individual strains to be transformed per day per robot; thus a whole yeast genomic library can be transformed in 6 days. In addition, our method provides an alternative approach to SGA methods for generation of combinatorial mutant yeast libraries.

## Materials and methods

### Liquid handling robot setup (S1–S5 Videos)

Our protocol was implemented using a Biomek FX liquid handling robot. A detailed mechanistic description of the pipetting, agitation and transfer operations undertaken by the robot, and associated programming is provided in the S1 File. The majority of brands of liquid handling robots available on the market (Hamilton, Tecan) can implement and operate the program described in the S1 File.

#### Yeast strain and growth conditions for liquid-handling robot manipulation (S1 Video)

Day 1: Up to 12 96-well yeast gene knockout library plates [2] were thawed and pinned into standard sterile 96-well plates with 200 μl YPD liquid medium by a Singer ROTOR robot or sterilized prongs for the purpose of growing starter cultures. Yeast were cultured on a rotary shaker at 200 rpm, 30°C for 24 hours. In addition, a number of 2.5 ml deep-well plates (VWR, Cat. No. 37001–520) equal to the number of starter culture plates were filled with one stainless steel bead (Biospec, Cat. No. 11079132ss) per well, and autoclaved for the following day.

Day 2: All plates including deep-well plates, read plates, selective media plates and the correlated tips are loaded at this step as well.

#### Inoculation and measurement of the OD_600_ (S2 Video)

800 μl of YPD medium was added to the deep-well plates using a multichannel pipette, liquid handling robot or a microplate dispenser such as a Matrix WellMate (Thermo scientific). 150 μl of cultured yeast cells were then inoculated into the deep-well plates using a liquid handling robot. We utilized a Biomek FX, however other liquid handling robots by manufacturers such as Tecan and Hamilton are also suitable (see S1 File).

Plates were briefly vortexed (600–1000 rpm, 1min) before the OD_600_ of each yeast culture was measured using a plate reader (e.g. BioTek Synergy 2). If the value was too low in some wells, more cells were inoculated to make the OD_600_ between 0.4–1.2. Note that the starting OD_600_ value for the growth step is fully customizable. For instance, the liquid handling robotic software we developed (described in our supporting information) can normalize all wells to the same OD_600_ value. Additionally, cells used to measure the OD_600_ value can be re-transferred back to the deep-well plate using the liquid handling robotic protocol (S1 File, S1–S5 Videos) in order to achieve a desired specific volume/cell density. The deep-well yeast plate was then transferred to the shaking incubator (Liconic, Cat. No. STX44-SA), which is connected with the Biomek FX to culture for another 2 ½ -4 hours (1200 rpm, 30°C).

#### High-throughput transformation preparation

During the 2 ½ -4 hours incubation time, the following was prepared:

1. 96 well plates with 200 μl selective medium in each well for the transformants. Plate number should equal the number of yeast plates being cultured for transformation. Medium was added by a liquid handling robot, Matrix WellMate microplate dispenser (Thermo scientific) or multichannel pipette.
2. PEG (50%w/v) was aliquoted in 2.5 ml deep-well plates, at 100 μl per transformation plates in the transformation process. For example, for 12 transformation plates total, a volume of at least 1200ul is required (a 100ul excess was commonly added in case of minor pipetting errors)
3. Transformation mix (Table 1):

**Table 1:** Transformation mix.

Component	1 well (μl)
LiAc (1M)	15
ssDNA	20
Plasmid/DNA + Water	15
Total	50

As with PEG, these volumes would be scaled according to the number of plates being transformed (50ul of transformation mix per plate). A suitable amount of plasmid or PCR product for each transformation reaction was added (e.g. plasmid: ~400ng/well, PCR Product: ~4500ng/well—see optimization in Results section). As a validation of our method in generating gene knockout strains, we utilized the following primers to delete and verify the efficiency of deletion for the gene *VPS38* (Table 2).

**Table 2:** VPS38 deletion and check primers.

*vps38Δ*-Fwd	GAATTGATGGTTTTACCTATTAGGGATAGTAATCATAATTTAAAAATCCAGCTGAAGCTTCGTACGC
*vps38Δ* -Rev	CATGGAAAAGATTAAATGGCAGTCCAAAAGAGATTTTTGATTTTCAGTGCATAGGCCACTAGTGGATCTG
*vps38Δ* -Check-A	GGAAACTCATAATGCTGGCAT
*vps38Δ* -Check-B	CTGCAGCGAGGAGCCGTAAT
*vps38Δ* -Check-C	CGACATCATCTGCCCAGATG
*vps38Δ* -Check-D	AGTGTGGATGTCGTCAGTTG

#### Read the OD of a plate after incubation (S3 Video)

After 2 ½-4 hours the deep-well yeast culture plates are removed from a shaking incubator, and the OD_600_ value is read. A range of OD_600_ 0.5–1.5 is optimal (see Results).

#### Switch to transformation (S4 Video)

When all the cells are ready to transform, all the deep-well plates from the incubator are transferred to the microplates “hotel” (Thermo Scientific Cat. No. 51021435). The temperature of the incubator will be shifted to 42°C.

#### Transformation (S5 Video)

The deep-well plates are centrifuged at 1500g for 5 mins and then placed back on the liquid handling robot, previously loaded with a rack of filter tips (200 μl filter tips (Axygen, Cat. No. FXF-180-R-S), 50 μl filter tips (Beckman Coulter, Cat. No. A21586), PEG and transformation mix solution plates (see high throuput transformation section above). The liquid handling robot then removes the YPD medium from the yeast culture plates, adds 50 μl Transformation mix, vortexes the plates at 600 rpm for 1 minute, adds 100 μl PEG to each well and vortexes for 1 minute. In total, each 96-well transformation plate well utilizes two unique tips (50 and 200) for the whole process. In contrast, only one set of tips for dispensing the PEG solution and transformation mix are required, since no direct contact with the yeast culture is made during dispensing. Together, this protocol essentially eliminates the possibility of contamination between wells, and the possibility for human error.

96-well plates now containing yeast, PEG and the transformation mix were returned to the shaking incubator at 42°C for 1–6 hours. Following completion of these steps for a single 96-well plate, the process was repeated for any additional yeast culture plates, which were all transferred automatically from the shaking incubator to the microplates “hotel” after incubation. All tips are also loaded onto the liquid handling robot from the connected microplate hotel. In total, 15 minutes of time on the liquid handling robot is required per plate transformation prior to the heat shock step. The number of plates transformed in a day will thus affect the minimum period of heat shock; for example, transforming 8 plates will necessitate a minimum 2 hour heat shock period (15mins x 8).

Transformation plates are removed from the incubator after heat shock, and centrifuged at 1500g for 5 minutes. Plates are then returned to the Biomek FX, which removes excess media, then transfers 30μl of concentrated transformed cells into a 96-well plate with appropriate selective media. In total, these post-heat shock steps require 15 minutes to complete, thus 12 plates could be finished in about 3 hours. In summary, 6 hours of liquid robotic handling time is utilized for 12 96-well plate transformation (1152 strains). Adding in initial growth time (2 ½ -4hours), the entire process takes between 8–10 hours (i.e. 1 day).

Selective media plates are incubated at 30°C for 2–4 days on a shaker (200 rpm). To prevent well evaporation, especially at the edge of the plates, parafilm wrapping and an incubation in a humid box is recommended. The cells are then spotted on selective meida plates, ideally by using the Singer ROTOR robot to pin the cells (Singer Instruments); multichannel pipettes and prongs also work but risk human error.

### Yeast microscopy and analysis

These methods have been described previously [26]. Briefly, stationary phase cells (OD_600_ >3.0) expressing pRB1 plasmid (Table 3) were examined using a Deltavision Elite microscope via a 100x objective. The data was analyzed by Fiji [28].

**Table 3:** Strains and plasmids used in this study.

Name	Genotype	Reference/Source
Strains		
BY4741	*MATa his3Δ1 leu2Δ0 met15Δ0 ura3Δ0*	
Plasmid		
pRB1	Pab1-GFP, Dcp2-mCh; Cen; URA3 marker	[27]

### Canavanine mutation frequency assay

To determine the mutagenic potential of differing lengths of 42°C heat shock, a canavanine resistance assay was utilized [29,30]. Briefly, wild type (WT) BY4741 cells were cultured to an OD_600_ of ~0.6 and then subject to our transformation method with differing 42°C incubation times (0, 1, 3, 6 hours). For each reaction, 1/200th of total cells in a transformation reaction were plated onto YPD plates for colony counting (dilution avoids formation of lawns). The rest of the cells are plated onto the canavanine plates (60mg/L, Sigma-Aldrich, Cat. No. C-9758). Both plate sets were incubated for 2 days at 30°C. The colony number was counted on both plate types, and the relative mutation frequencies of yeast subject to differing 42°C heat shocks were calculated.

## Results

### ≥100ng of plasmid is suitable for high-throughput transformation of yeast over a broad OD_600_ range

**Fig 1:**
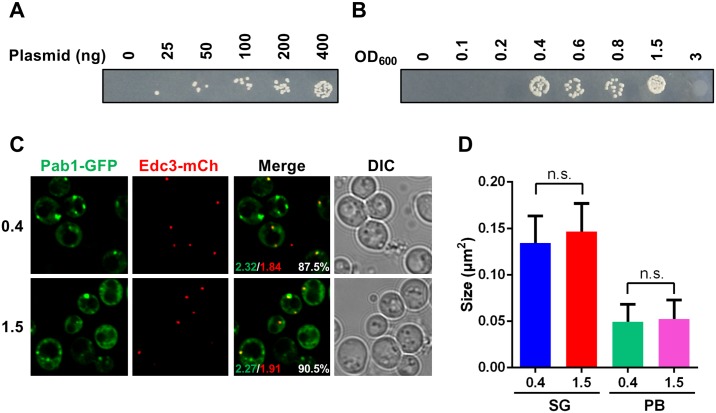
Plasmid and cell concentration affect transformation efficiency. (A) BY4741 cells at OD_600_ 0.5 were transformed with the indicated amount of pRB1 plasmid using our automated methodology with 4-hour heat shock. After transformation, cells were spotted on a –Uracil agar plate. (B) Different OD_600_ cells were transformed with 100ng pRB1 plasmid and heat shocked at 42°C for 4 hours. (C) Microscopy of BY4741 cells originally transformed either at 0.4 or 1.5 OD_600_, and examined at an OD of 3.5. Numbers indicate average stress granule (Pab1-GFP, green text) or P-body (Edc3-mCh, red text) foci per cell, and the percentage of Edc3-mCh foci co-localized with Pab1-GFP (white text) (D). Quantification of stress granule (SG) and P-Body (PB) size in (C). Data is presented as mean ± standard deviation of 3 independent experiments; n.s., not significant.

To optimize our high-throughput transformation methodology using a liquid handling robot, the effect of varying plasmid and cell concentration was tested. First, to assess the importance of plasmid concentration, pRB1, a single-copy *URA3* based plasmid expressing Pab1-GFP and Edc3-mCh was transformed into BY4741 WT yeast cells at differing concentrations, followed by selection for transformants on media lacking uracil. Pab1 is a poly(A)-binding protein that aids mRNA translation, while Edc3 is a mRNA decay factor. Pab1 and Edc3 are also cytoplasmic markers of conserved mRNA-protein (mRNP) foci called stress granules (Pab1) and P-bodies (Edc3), which are implicated in regulation of mRNA function [31]. As expected, at a cell density of 0.5, transformant numbers increased as plasmid concentration in the transformation reaction increased from 25ng to 400ng total (Fig 1A).

To assess the importance of cell density in our methodology, 100ng of pRB1 was transformed into yeast cells harvested at a range of cell densities (Fig 1B). At low concentrations (OD_600_<0.2), no transformants were observed. However, efficient transformation was observed at a broad OD_600_ range, from 0.4 to 1.5 (Fig 1B). Harvesting cells at a high OD_600_ (3.0) cells did not generate transformants, indicating that post-diauxic shift yeast are not suitable for high-throughput transformation. In summary, an OD_600_ range of 0.4–1.5, and >100ng plasmid DNA is optimal for our automated transformation method.

We next examined whether high OD_600_ transformed cells exhibited any difference in plasmid expression or general cellular phenotypes, which we assessed by any changes in cell morphology or induction of stress granules and P-bodies relative to low OD_600_ transformed cells. Stress granules (Pab1-GFP foci) and P-bodies (Edc3-mCh) were induced by growth to stationary phase (OD_600_~3.5), which results in robust induction of both granules, as well as their partial co-localization in the cytoplasm [27]. As shown in Fig 1C and 1D, there was no obvious difference in cell morphology. Additionaly, no difference was observed in the co-localization between stress granules and P-bodies, or in the average size or number of stress granules and P-bodies per cell. In summary, this indicates that no obvious problems result from transformation at a range of cell densities.

### Increasing heat shock up to 6 hours increases transformation efficiency with minimal effects on mutational frequency

**Fig 2:**
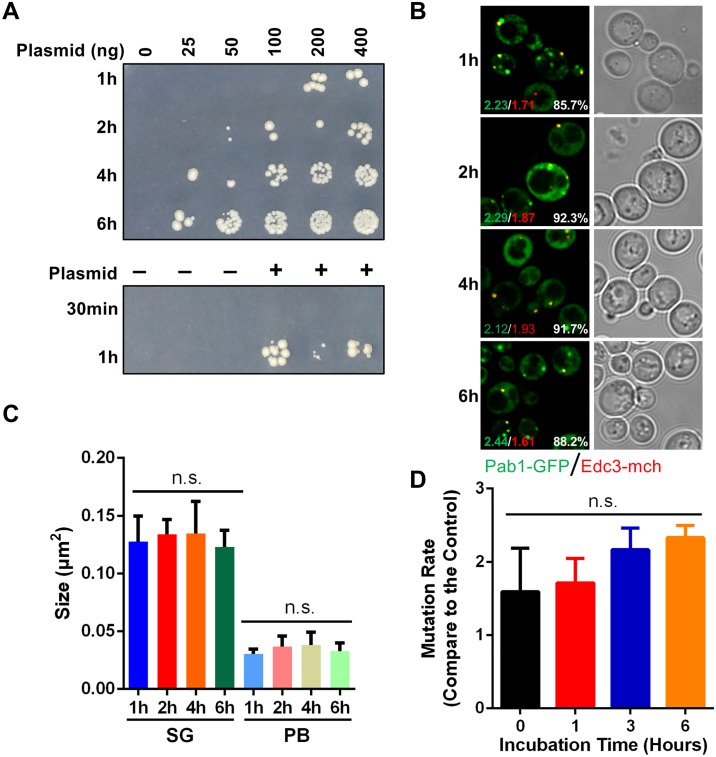
Transformation efficiency is increased with extended 42°C heat shock periods. (A) BY4741 cells at OD_600_ 0.5 were transformed with the indicated amount of pRB1 plasmid and incubated at 42°C for 30 minutes (min), 1, 2, 4, and 6 hours (h). Transformants were selected on –Uracil plates. (B) Transformed cells generated from transformation reactions with different 42°C incubation times (1, 2, 4 and 6 hours). Numbers indicate average stress granule (Pab1-GFP, green text) or P-body (Edc3-mCh, red text) foci per cell, and the percentage of Edc3-mCh foci co-localized with Pab1-GFP (white text). (C) Quantification of stress granule (SG) and P-body (PB) size in (B). Data is presented as mean ± standard deviation of 3 independent experiments; n.s., not significant. (D) BY4741 cells transformed with pRB1 plasmid and incubated at 42°C for different time were assessed for elevated mutation rates by development of canavanine resistance. Transformed cells were plated on canavanine media (60 mg/L). Simultaneously, 1/200 diluted amount of cells were coated on the YPD plate. Colony number was counted after 2 days incubation at 30°C. Mutation rates were normalized to the 0-hour heat shock. Data is presented as mean ± standard deviation of 3 independent experiments; n.s., not significant.

The next variable we optimized for our automated transformation method was the 42°C heat shock duration (Fig 2A). Surprisingly, a 30 minutes incubation resulted in no transformants in our experimental conditions; this may reflect a shorter 30°C or room temperature incubation time of the yeast in the transformation mix relative to other described methods [32,33] where 30 minutes at 42°C does generate transformants. However, significant transformant numbers were observed with extended incubation times (1–6 hours) at 42°C, with maximal transformant numbers observed with a 6 hour heat shock. Consistent with Fig 1A, transformant number was also increased by increasing plasmid concentration for all transformation reactions with differing 42°C periods.

Since 6 hours is considerably longer than that associated with current yeast transformation methods [23,24], we were curious to see if this had any detrimental effects to yeast. First, general cell morphology and pRB1 plasmid expression was again examined by microscopy. No obvious differences in cell morphology, Pab1 and Edc3 expression levels, and stress granule and P-body formation/co-localization were observed between cells subject to different 42°C transformation periods (Fig 2B and 2C). Second, we assessed the effects of extended heatshock on the accumulation of spontaneous genomic mutations, by using a canavaine resistance assay [29,30]. Briefly, *CAN1* is a gene that encodes an arginine permease. Canavanine, a toxic arginine analog, cannot be taken up in cells harboring deficient *CAN1*. Thus, cells harboring a WT *CAN1* cannot grow on canavanine plates, whereas yeast that have acquired inactivating mutations in *CAN1* do grow. We measured *CAN1* mutation rates and found there was no statistically significant difference in relative mutation rates for cells incubated at 42°C for between 1–6 hours (Fig 2D). As expected, cell viability decreased with longer heat shock periods in BY4741 cells (data not shown), but this did not preclude obtaining transformants (Fig 2A). Taken together, these data demonstrate that a wide array of 42°C heat shock time periods can lead to efficient transformation rates, thus offering considerable flexibility to the implementation of our protocol.

### Automated transformation protocol is a viable method for generation of combinatorial yeast mutant libraries

**Fig 3:**
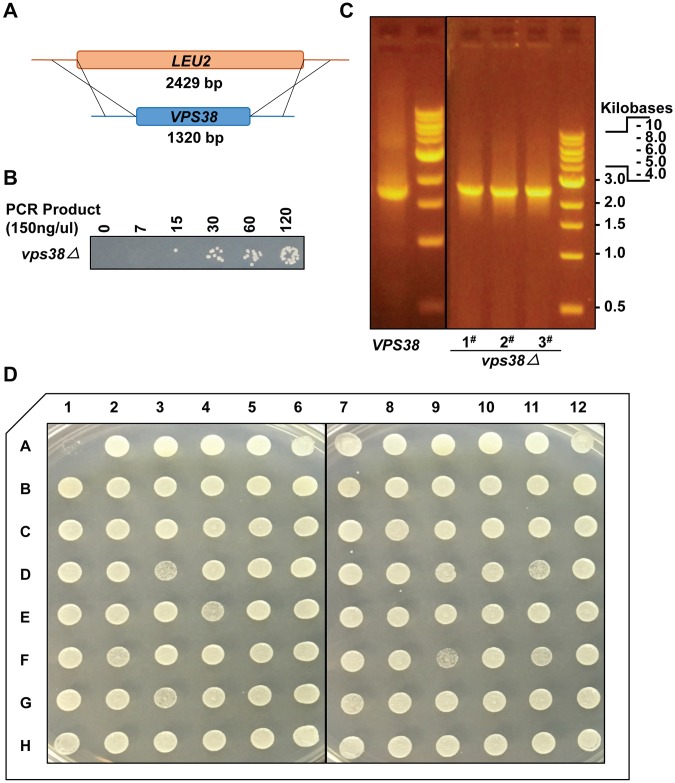
Successful application of high-throughput transformation method to gene deletion. (A) Generation of *VPS38* knockout strain by insertion via homologous recombination of a *LEU2* selective marker at the *VPS38* locus. (B) Different volumes of 150ng/ul of the *LEU2* cassette were transformed into yeast cells. Transformants were plated on—Leucine media. (C) *VPS38* deletion was verified using check primers (Table 2) from four single colonies (wild type, 1#: 30 μl spot, 2#: 60 μ spotl, 3#: 120 μl spot). Expected PCR product size of wild type: 1701 bp, *VPS38* knockout: 2810 bp. (D) Plate 1 from the non-essential yeast knockout library was cultured and transformed with 4500ng VPS38 knockout *LEU2* cassette using our methodology. Transformed cells were cultured in –Leucine SD media for 3 days and spotted on –Leucine media using prongs.

Transformation of a linear DNA construct and manipulation of genomic sequence via homologous recombination is a well-established yeast methodology [34,35]. We were thus curious if our method could be utilized for semi-high throughput construction of combinatorial yeast gene deletion libraries via transforming specific gene deletion PCR product, akin to the result of synthetic genetic array methodologies. To test this, we generated via PCR a *LEU2* cassette (Fig 3A) with 45 nucleotide ends homologous to sequence flanking the *VPS38* ORF, which is a non-essential gene. We then tested transformation of different concentrations of the *LEU2* cassette into BY4741 cells using our automated transformation method. After 3 days growing in the selective medium, cells were plated on –Leucine selective media. As expected, more transformants were observed with increasing amounts of the *LEU2* cassette (Fig 3B). Wild type cells and cells from three separate colonies transformed with different amount of the *LEU2* cassette (30, 60 and 120ul 150 ng/ul PCR product; concentrated to a final volume of 10ul by a speedvac) were cultured, genomic DNA isolated, and the *VPS38* locus assessed for the presence or successful deletion of the *VPS38* gene using primers flanking the homologous recombination sites (Fig 3C). All 3 colonies which were examined showed successful deletion of *VPS38*.

Finally, we demonstrated the applicability of our method to high throughput transformation of gene deletion library plates, wherein each well harbors a unique yeast gene deletion strain, some of which exhibit lower growth rates than WT/other gene deletion strains, thus making transformations potentially more challenging. Cells in the A1 well were removed prior to initial culture to act as a negative control. Importantly, we obtained 100% successful transformation of all strains, following growth to OD_600_ ~0.8, transformation with 4500ng total *LEU2* cassette and incubation at 42°C for 3 hours. This demonstrates the effectiveness of our method in generating combinatorial gene mutant libraries in yeast.

## Discussion

Based on it’s simple genome and high degree of conservation to human cells, yeast is an excellent model to study fundamental biological processes, and to rapidly screen for genetic modifiers of pathology observed in various human diseases [7,15]. High-throughput transformation is an important tool for genetic screening in yeast, and here we describe a high-throughput automated method that efficiently transforms plasmid or linear DNA constructs into yeast.

**Fig 4:**
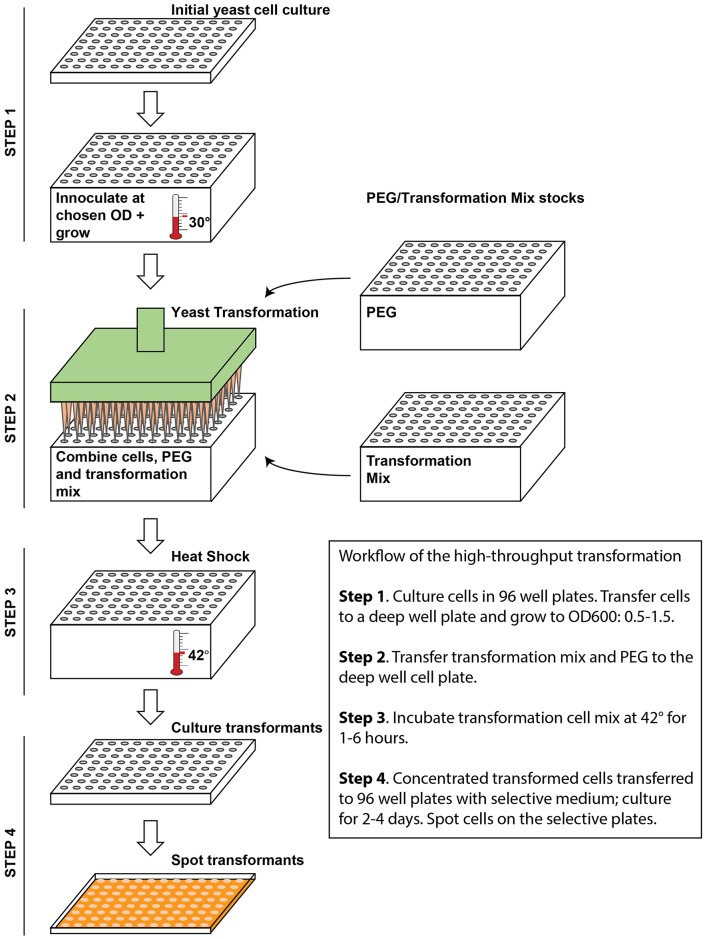
Schematic model of yeast transformation using liquid handling robot. Step 1, Inoculate overnight culture to the deep well plate. Grow another 2 ½ -4 hours to recover cells to the mid-log phase. Step 2, Prepare plasmid and transformation mix to transformation. Normally, an OD_600_ range of 0.4–1.5, ≥100ng plasmid or 4500 ng PCR product is optimal for our automated transformation method. Step 3, Heat shock of the transformants for 3–6 hours. Step 4, Transfer the transformed cells to a liquid selective media plate to grow another 2–4 days. Pin the transformed strains onto appropriate selective media to generate the new library.

Here, we have taken a previously published microtiter plate transformation method [23,24], and adapted it to a liquid handling robotic platform. In the process, we optimized key factors affecting transformation efficiency, such as plasmid amount, cell concentration and 42°C incubation time. Additionally, we demonstrated its efficiency in plasmid transformation and gene deletion, making this method highly useful for genome-wide genetic analyses (Fig 4, S1–S5 Videos). We believe this is a useful advancement for the yeast community for the following reasons.

First, use of a liquid handling robot essentially eliminates the possibility of human error if doing microtiter plate transformations by hand. Second, less time is spent doing a transformation reaction using the automated approach. Third, the throughput of the automated transformation method is far greater over a manual approach, with 12 plate transformations/day a realistic goal per liquid handling robot. These and other basic transformation advantages are highlighted in Table 4.

**Table 4:** Comparison of the two plasmid transformation methods.

Plasmid Transformation		
	Gietz and Schiestl Method	This Method
Cell concentration (OD_600_)	Not specified	0.4–1.5
Plasmid Concentration (ng)	Not Specified	≥100
Heat Shock Time	1 hour	1–6 hours
Scale	1 microtiter plate	Unlimited
Operation	Manual	Automatic
Error Rate	High	Low

In addition, our method offers a potential alternative method to SGA approaches for generating combinatorial yeast mutant libraries. Key advantages are summarized in Table 5. Additional benefits include being non-reliant on meiotic recombination events inherent in the SGA approach, which can lead to potential complications in obtaining double mutants when both genes are proximal on the same chromosome, or the loss of selective markers up or downstream of a given modified gene of interest if a recombination event occurs between them [36].

**Table 5:** Comparison of SGA and high-throughput gene deletion methods.

Gene Deletion		
	SGA	This Method
Query strain construction	Yes	No
Number of different selective plates used	6	1
Time to generate one microtiter plate’s worth of knockout strains	About 18 days	6 Days
Handling time for one microtiter plates	7 times (15 minutes each)	30 minutes
Complexity of process	Normal	Easy

However, a caveat of our method that limits its applicability to whole genome analysis is the need to verify that gene modification/knockout has occurred at the correct locus with a given selection cassette. One viable approach to rapidly isolate yeast that have been correctly deleted for a gene of interest is to spot and serially dilute transformed yeast cells following 1 day of growth in selective media, such that 1–5 single colonies grow on the agar plate. These individual colonies can then be inoculated into liquid media by the Singer Rotor robot (or manually), and subject to a colony PCR protocol. Finally, PCR verification using primers to ensure correct integration of the KO cassette at the correct genomic and gel electrophoresis analysis, ideally using a high-throughput gel apparatus such as GE Healthcare, Ready-To-Run Unit and Thermo Scientific, E-Gel 96 system, will allow fast conformation as to which double mutant strains have been successfully constructed. Should PCR indicate a mixed population of correct and incorrect strains, streaking for single colonies and repeating the above process would be required. For this reason, we believe our method is best suited to creating targeted collections of double mutants in the 100–1000 strain range, rather than entire genomic libraries.

To ensure maximal efficiency of correct insertion of any deletion cassette, it is good practice to utilize selectable markers for which no homologous sequence exists in yeast, and maximize the nucleotide length of sequence homologous to the target of interest. Generally, 45nts results in 80% insertion at the correct locus, whereas 60nts results in 90% correct insertion [37].

Another significant benefit of our method is the reduced use of consumables. Every individual transformed strain uses two tips (one 200 μl, one 50 μl) for the whole process including inoculation, measuring of cell concentration and the transformation reaction itself. However, only a single rack of tips is utilized for addition of the transformation mix and PEG mix for *all* plates transformed. In contrast, a manual approach would utilize a new rack of tips for PEG and transformation mix for every transformed plate. Hence, 12 plates transformed by our method needs 14 racks of P200 and 12 racks of P50 tips, while manual transformation needs 2–4 times as many tips with a greater risk of human error. In addition, in comparison to a SGA methodology, which utilizes 6 different types of selective media plates, our methodology only uses 1 type of final selective plate to generate combinatorial mutants.

There are many potential applications of our method, depending upon the library of strains transformed, and the nature of the foreign DNA (plasmid or genome-modifying cassettes) introduced. For example, genome scale transformation of a plasmid bearing a tagged protein of interest into an alternatively tagged ORF genomic library (e.g. TAP-tag collection [5] could allow genome-wide interaction studies (e.g. Co-IP). Transformation of an RFP-tagged protein of interest into the yeast GFP library [38] could allow genome-wide co-localization studies. Analogous to SGA analysis, generation of combinatorial yeast mutant strains could also help elucidate complex genetic networks and identify enhancers or suppressor of a given gene of interest, including heterologous gene products implicated in human disease.

In our method, a Biomek liquid handling robot from Beckman Coulter was used, however we are keen to stress that a variety of liquid handling robots from different manufacturers, such as Hamilton, Tecan, and others can also be adapted to our method. We are happy to advise in this matter if necessary. In summary, our method provides an efficient and inexpensive way to conduct high-throughput genetic network and functional analyses in yeast.

## Supporting information

S1 File Supplemental methods for robot setup and video information. (DOCX)

S1 Video Deckload. This is the first module of the Transformation Method. It allows the operator to load new plate sets. Each set contains an overnight culture plate, a deep-well growth plate, a clear bottom plate to read the OD, a plate of selective media for the final transformed yeast, a set of 50 μl tips, a set of 200 μl tips, and a plasmid plate, unless 1 plate of plasmids is being used for all plates. The operator is required to direct the method to a csv file with the names and barcodes of the overnight culture plate, selective media, and the plasmid plate if individual plasmid plates are being used. This information is used for barcode verification of these plates while they are being loaded so that there cannot be any mix-ups of which overnight culture plate will be transferred into which selective media plate at the end of the method. The sets are loaded into the robot, and accessed by the robot in a fashion that ensures that all of the members of the plate set are used together and that there is no risk of parts of one set being confused with another set.
(MP4)

S2 Video Inoculation. This module of the Transformation Method allows the operator to inoculate the deep-well growth plate from the overnight culture. After inoculation the operator may read the OD of the growth plate and choose to inoculate again to reach a higher OD, or load the data from the OD into the method and have the robot normalize all of the wells on the plate to the highest OD or an OD of the operator’s choosing.
(MP4)

S3 Video Read OD. This module allows the operator to read the OD of any of the deep-well growth plates. The culture is transferred into a clear bottom plate for the reading and then transferred back after the reading is completed so that none of the volume is lost. This allows the operator to read the OD as many times as is necessary without concern of depleting the culture.
(MP4)

S4 Video Switch to transformation. When the operator confirms that they wish to perform the transformation, the robot changes into Transformation mode, the growth plates are moved to the ambient hotel and the incubator is changed to 42°C.
(MP4)

S5 Video Transformation. This module of the Transformation Method allows the operator to transform plates. The operator places a deep-well plate of PEG, a deep-well plate of plasmid (unless different plasmid plates are being used for each transformation), and additional tips on the robot deck. The robot then brings out the deep-well plate of yeast and prompts the operator to centrifuge it. The robot then decants off the growth media, adds the PEG, adds the plasmid, and puts the plate in the incubator (at 42°C). After the plate is in the incubator, the operator has the option to transform another plate while the first plate is incubating or to wait for the first plate to be ready. When each plate has incubated for the desired time, the robot alerts the operator. Once the operator confirms that they are ready to continue the robot brings out the plate and prompts the operator to centrifuge the plate. When the operator returns the centrifuged plate to the robot, the robot decants off the supernatant, mixes the remaining pellet, and transfers the transformed yeast into a plate of selective media.
(MP4)
