# Supplementary material for: High-throughput transformation of Saccharomyces cerevisiae using liquid handling robots
Source: PLoS One. 2017 Mar 20;12(3):e0174128. doi: 10.1371/journal.pone.0174128 (PMC5358765; doi:10.1371/journal.pone.0174128)
Supplement: S1 File — (DOCX) [file pone.0174128.s001.docx]

# Yeast Transformation Method using Liquid Handling Robot

## Method Overview

This method was written to be run on a dual bridge Beckman Coulter Biomek FX with integrated Liconic STX-44 incubator, Cytomat 6000 series ambient microplate “hotel” (Thermo Scientific Cat. No. 51021435), orbital shaker ALP (Beckman Coulter, Cat. No. 379448), and shaking peltier ALP (Beckman Coulter, Cat. No. A93942). Similar equipment from Tecan and Hamilton is also suitable for this purpose.

This robot method has been written in a modular fashion with all of the modules inside a loop that allows the operator to select which function they wish to perform at nearly any time. The operator can select to “Load new plate sets”, “Inoculate a plate”, “Read the OD of a plate”, “Do transformations”, “Unload a plate set”, “Recover from a crash”, or “Stop the method”.

The method operates is two modes; “Grow“, and “Do Transformations”. In Grow mode, cells are cultured in the incubator at 30^o^, with shaking at 1200 rpm, and the operator can select any of the modules. Once “Do transformations” is selected the cultures of yeast growing in the incubator are transferred to the Cytomat storage hotel, and the incubator is set to 42^o^. This is an irreversible change. Once the method has switched to the “Do Transformations” mode, the operator can no longer load new plates, nor inoculate, nor read the OD.

The method operates with preset default values for all of the transfer volumes and incubation times, but the operator is given the opportunity to change these values at the beginning of each module each time.

## Load new plate sets (S1 Video)

This robot method is able to run from 1 to 14 plate sets at a time. A plate set consists of: an overnight culture plate (300 ul, sterile, flat bottom – e.g. Genesee Scientific, Cat. No. 25-104) a deep-well growth plate (2.2 ml, sterile, square well plate – e.g. VWR, Cat. No. 37001-520) with media and an agitation bead (Biospec, Cat. No. 11079132ss), a selective media plate (same as overnight culture plate), a read plate (300 ul, sterile, clear bottom e.g. Genesee Scientific, Cat. No. 91-420TB), a plasmid plate (200 ul, sterile, conical well), and a rack of P50 (Beckman Coulter, Cat. No. A21586) and P200 (Axygen, Cat. No. FXF-180-R-S) aerosol resistant tips. The operator may load all of the sets for the day at once or continue to load sets as the cultures are growing. The operator may also choose to use one plasmid plate for all of the transformations (deep-well plate) or use a separate plasmid plate (see above) for each yeast plate. The plasmid plates may be loaded during the loading sequence or the method will prompt the operator to provide them during the transformation step.

To load a plate set, or multiple sets the operator is prompted to select a worklist file with the barcodes for the overnight culture plate, plasmid plate, and the selective media plate. To eliminate the possibility of plate inversions or swaps, all of the input plates (overnight culture and plasmid plates) and output plates (selective media plate) are barcoded on the front of each plate. The method then prompts the operator to:

- Load the plates on the robot deck
- Scan barcodes of plates
- The robot files the plates and tips into the microplate hotel
  - Growth plate into the Liconic incubator 30^o^,1200 rpm)
  - All other plates and tips into Cytomat hotel

## Inoculate a plate and read the OD_600_ (S2 Video)

The operator may choose to inoculate the plate and return everything to the hotels or to inoculate the plate and immediately read the OD. If the operator reads the OD they then have the opportunity to perform the inoculation again with the 96 tip head, or provide an additional rack of tips and use the 8 tip head to normalize the plate to the highest OD600. The inoculation sequence goes as follows:

- The operator is able to select from any plate set that has been loaded
- The overnight culture, growth, and optical plates and a set of P50 and P200 aerosol resistant tips are unloaded from the storage hotel and incubator to the robot deck.
  - The overnight culture is moved to the orbital shaker and shaken at 1000 rpm for 60 seconds
  - The deep-well growth plate is loaded onto the shaking peltier position (at 30^o^ shaking at 1000 rpm)
- Using the P50 tips the overnight culture is mixed by pipetting 39 ul up and down 6 times
- 100 ul (default volume) of overnight culture is transferred to the growth plate containing 1000 ul (default volume) of YPD.
  - If the transfer volume is greater than 45 ul it is divided up into a series of 45 ul transfers and then the remainder volume is transferred
- The deep-well growth plate is mixed by pipetting 39 ul up and down 6 times
- The deep-well growth plate is moved from the shaking peltier position to the orbital shaker and shaken at 900 rpm for 20 seconds
- The growth plate is moved back to the shaking peltier position
- If the operator has selected to read the OD_600_ after inoculation, the following sequence is conducted:
  - Using the P200 tips 200 ul (default volume) is transferred from the deep-well growth plate to the optical read plate
    - If the transfer volume is greater than 150 ul it is divided up into a series of 150 ul transfers and then the remainder volume is transferred
    - This transfer required a custom pipetting template. To ensure that the tips would not crash into the agitation bead in the growth plate, the tip descends into well at 25% speed. At 4.6 mm from the bottom, it moves 100% of the allowable distance into the back right corner of the well, then continues down to 2.6 mm above the bottom and then slows its descent to 5% speed and continues to 0.6 mm from the bottom where it aspirates the volume at 20 ul/sec. This movement allows the tip to push the agitation bead to the side and aspirate from the bottom of the well.
  - The operator reads the OD on the BioTek Synergy 2 plate reader (other equivalent OD readers are also suitable).
  - The operator returns the optical read plate to the robot deck
  - Using the P200 tips 200 ul (default volume) is transferred back to the media plate
    - If the transfer volume is greater than 150 ul it is divided up into a series of 150 ul transfers and then the remainder volume is transferred
    - This transfer required a custom pipetting template. To ensure that the yeast would not be left around the well edge, the transfer volumes were split in 40 moving aspirations, each aspiration moves 10 degrees of the perimeter of the well aspirating 1/40 of the volume. This was followed by an additional 100 ul transfer of the same style to be sure that there was no remaining volume.
  - If the operator wishes to have a higher starting OD they have the option to perform the inoculation steps again
    - If this is selected the above sequence is repeated
  - If the overnight cultures provide an irregular series of OD_600_ inoculations, the operator can choose to select "I'd like to use the 8 channel to normalize the plate ODs"
    - The method will prompt the operator to select the OD_600_ output file from the plate reader
    - The method will use the OD_600_ values, growth plate well volume, and inoculation volume to calculate the OD_600_ of the overnight culture wells and will calculate the additional inoculation volume necessary to bring each well up to the highest OD_600_ on the plate
      - The operator is given the option to replace the highest OD_600_ value with a value of their choosing
    - The operator will be prompted to supply an additional rack of P50 aerosol resistant tips for the normalization
- When the inoculation sequence is complete the plates and tips are returned to their original locations in the incubator and microplate hotel

## Read the OD of a plate after incubation (S3 Video)

- The operator is able to select from any plate set that has been loaded into the robot
- The Read the OD sequence is described above as an option in the inoculation routine

## Transform a plate

## Switch to transformation (S4 Video)

- The operator is asked to confirm this choice because it is an irrevocable selection
- The robot moves all of the growth plates from the incubator to the Cytomat hotel and sets the incubator to 42^o^

**Transformation (S5 Video)**

- The operator is able to select from any plate set that has been loaded into the robot
- The operator is prompted to place a deep well plate of PEG mix, a deep well waste plate, a plasmid plate and an additional rack of P200 aerosol resistant tips on the deck
- The robot moves the growth plate and a rack of P200 aerosol resistant tips from the Cytomat hotel onto the deck.
- The operator is asked to remove the deep-well growth plate from the deck and spin it for 5 minutes at 1500 g at 20^o^, then return it to the deck.
- Using the P200 tips, 1000 ul (default volume) of the supernatant is transferred from the growth plate to the waste plate
  - If the transfer volume is greater than 150 ul it is divided up into a series of 150 ul transfers and then the remainder volume is transferred
  - This transfer required a custom pipetting template to ensure that the tips would not crash into the agitation bead in the growth plate. This technique is described above in the inoculation routine.
- Using the P200 tips, 50 ul (default volume) is transferred from the plasmid plate to the growth plate
- Using the P200 tips, 100 ul (default volume) is transferred from the PEG mix plate to the growth plate
  - This transfer required a custom pipetting template due to the viscosity of the PEG mix. The dispense step is performed in stages. The full volume is dispensed in the air above the volume at 20 ul/sec, then an equal volume of air is immediately aspirated at 200 ul/sec, the there is a 0.4 sec pause that allows the residue to settle down in the tip of the pipette tip, then the volume is dispensed a second time at 20 ul/sec, and 50% of the volume of air is aspirated at 200 ul/sec, there is a second 0.4 sec pause for the volume to settle, and then the 50% volume is dispensed at 20 ul/sec.
- The PEG volume is then mixed into the samples with a series of 4 aspirations and dispenses using the technique that avoids the agitation beads (described above) with the aspiration height set to 0.6 mm above the bottom of the well.
- The plate is then transferred into the incubator at 42^o^
  - The time that the plate is transferred into the incubator is recorded and stored in a data array. The operator has the option to perform the transformation steps on additional plates during the incubation period (their incubation start times will be individually stored).
- Once the incubation is complete (60 minute default) the robot retrieves the plate and returns it to the deck and the operator is prompted to centrifuge the plate for 5 minutes at 1,500 x g.
- The supernatant is then transferred to the waste plate with 2 transfers of 150 ul using the technique that avoids the agitation beads (described above) with the aspiration height set to 0.8 mm above the volume of the well.
- Using P50 tips, the pellet is then mixed into the samples with a series of 4 aspirations and dispenses using the technique that avoids the agitation beads (described above) with the aspiration height set to 0.6 mm above the bottom of the well.
- Using the same P50 tips, 5 ul (default volume) is transferred to the selective media plate. Increasing the volume transferred may increase the likelihood of obtaining successful transformants, particularly in sick strains.
- The operator is then prompted to remove the plates and tips from the deck. If additional plates are being transformed the method will retrieve them and perform the steps described above when their incubation is complete.

## Unload Plate Sets

Once the method is complete the robot will retrieve all of the remaining items from the Cytomat hotel and prompt the operator to clear the deck as needed.

## End Method

- When to operator selects this option the robot will unload all sets of plates from the Cytomat ambient hotel and Liconic incubator and load them on the robot deck

**Video Information**

**Transformation Method Overview**

The transformation method is written in a modular fashion that enables the operator to use the robot in as flexible a fashion as possible. All of the plates and tips necessary to inoculate, grow, read the OD, and transform a plate make up a “plate set”. It allows the operator to load new plate sets, inoculate a growth plate, read the OD of a growth plate, unload a set of plates, or perform the transformation. The method functions in two modes; “Growth mode” and “Transformation mode”, due to the need to change the temperature of the incubator. In Growth mode the incubator is set to 30˚ and the load, inoculate, and read OD functions are available. Once the operator selects “Do Transformation” the mode is changed to Transformation mode, the growth plates are transferred to the ambient hotel, and the incubator is set to 42˚. Once the mode is changed to Transformation mode, the operator can only transform plates and unload plate sets.

**S1 Video-Deckload**

This is the first module of the Transformation Method. It allows the operator to load new plate sets. Each set contains an overnight culture plate, a deep-well growth plate, a clear bottom plate to read the OD, a plate of selective media for the final transformed yeast, a set of 50 µl tips, a set of 200 µl tips, and a plasmid plate, unless 1 plate of plasmids is being used for all plates. The operator is required to direct the method to a csv file with the names and barcodes of the overnight culture plate, selective media, and the plasmid plate if individual plasmid plates are being used. This information is used for barcode verification of these plates while they are being loaded so that there cannot be any mix-ups of which overnight culture plate will be transferred into which selective media plate at the end of the method. The sets are loaded into the robot, and accessed by the robot in a fashion that ensures that all of the members of the plate set are used together and that there is no risk of parts of one set being confused with another set.

**S2 Video-Inoculation**

This module of the Transformation Method allows the operator to inoculate the deep-well growth plate from the overnight culture. After inoculation the operator may read the OD of the growth plate and choose to inoculate again to reach a higher OD, or load the data from the OD into the method and have the robot normalize all of the wells on the plate to the highest OD or an OD of the operator’s choosing.

**S3 Video-Read OD**

This module allows the operator to read the OD of any of the deep-well growth plates. The culture is transferred into a clear bottom plate for the reading and then transferred back after the reading is completed so that none of the volume is lost. This allows the operator to read the OD as many times as is necessary without concern of depleting the culture.

**S4 Video-Switch to Transformation**

When the operator confirms that they wish to perform the transformation, the robot changes into Transformation mode, the growth plates are moved to the ambient hotel and the incubator is changed to 42˚.

**S5 Video-Transformation**

This module of the Transformation Method allows the operator to transform plates. The operator places a deep-well plate of PEG, a deep-well plate of plasmid (unless different plasmid plates are being used for each transformation), and additional tips on the robot deck. The robot then brings out the deep-well plate of yeast and prompts the operator to centrifuge it. The robot then decants off the growth media, adds the PEG, adds the plasmid, and puts the plate in the incubator (at 42˚). After the plate is in the incubator, the operator has the option to transform another plate while the first plate is incubating or to wait for the first plate to be ready.

When each plate has incubated for the desired time, the robot alerts the operator. Once the operator confirms that they are ready to continue the robot brings out the plate and prompts the operator to centrifuge the plate. When the operator returns the centrifuged plate to the robot, the robot decants off the supernatant, mixes the remaining pellet, and transfers the transformed yeast into a plate of selective media.
